# Prolonged Exposure to High Temperature Inhibits Shoot Primary and Root Secondary Growth in *Panax ginseng*

**DOI:** 10.3390/ijms231911647

**Published:** 2022-10-01

**Authors:** Jeongeui Hong, Kyoung Rok Geem, Jaewook Kim, Ick-Hyun Jo, Tae-Jin Yang, Donghwan Shim, Hojin Ryu

**Affiliations:** 1 Department of Biological Sciences and Biotechnology, Chungbuk National University, Cheongju 28644, Korea; 2 Department of Biological Sciences, Chungnam National University, Daejeon 34134, Korea; 3 Department of Herbal Crop Research, National Institute of Horticultural and Herbal Science, Rural Development Administration, Eumseong 27709, Korea; 4 Department of Agriculture, Forestry and Bioresources, Plant Genomics and Breeding Institute, College of Agriculture and Life Sciences, Seoul National University, Seoul 08826, Korea

**Keywords:** *Panax ginseng*, heat stress, RNA-seq, hormone, photosynthesis

## Abstract

High temperature is one of the most significant abiotic stresses reducing crop yield and quality by inhibiting plant growth and development. Global warming has recently increased the frequency of heat waves, which negatively impacts agricultural fields. Despite numerous studies on heat stress responses and signal transduction in model plant species, the molecular mechanism underlying thermomorphogenesis in *Panax ginseng* remains largely unknown. Here, we investigated the high temperature response of ginseng at the phenotypic and molecular levels. Both the primary shoot growth and secondary root growth of ginseng plants were significantly reduced at high temperature. Histological analysis revealed that these decreases in shoot and root growth were caused by decreases in cell elongation and cambium stem cell activity, respectively. Analysis of *P. ginseng* RNA-seq data revealed that heat-stress-repressed stem and root growth is closely related to changes in photosynthesis, cell wall organization, cell wall loosening, and abscisic acid (ABA) and jasmonic acid (JA) signaling. Reduction in both the light and dark reactions of photosynthesis resulted in defects in starch granule development in the storage parenchymal cells of the main tap root. Thus, by combining bioinformatics and histological analyses, we show that high temperature signaling pathways are integrated with crucial biological processes that repress stem and root growth in ginseng, providing novel insight into the heat stress response mechanism of *P. ginseng*.

## 1. Introduction

Plants are constantly exposed to a wide range of abiotic and biotic stresses, which negatively impact their growth, development, and productivity [1,2,3,4]. As sessile organisms, terrestrial plants must coordinate their physiological responses to adapt to the broad range of environmental stresses. The global temperature is expected to rise by 2–5 °C by the end of the century, owing to global warming [5,6]. This is especially concerning because an increase of 3–4 °C is expected to reduce crop productivity by 15–35% [7,8]. When exposed to extreme temperatures, plants suffer significant, and possibly irreversible, damage. Plants have evolved sophisticated hormonal and physiological pathways to respond to high temperature stress [9,10]. Therefore, to ensure food security under future climate scenarios, it is critical to investigate the high temperature response mechanisms and signaling pathways of plants.

Korean ginseng (*Panax ginseng* C.A. Meyer) has a long history of use as a valuable medicinal plant in Asian countries, particularly Korea, China, and Japan [11,12]. The roots of *P. ginseng* plants contain a variety of beneficial compounds, including ginsenosides, which exhibit a wide range of therapeutic effects, and boost the immune system and promote vitality in humans [13,14,15,16]. However, because of the restricted special cultivation features and limited genome information of *P. ginseng*, the genetic and physiological analyses of its growth and development have been challengeable [17,18,19]. *P. ginseng* is a perennial shade plant that exhibits hypersensitivity to high temperatures. Ginseng leaves exhibit growth retardation and typically burn after being exposed to 30 °C for longer than 5 days. The development and output of *P. ginseng* plants are seriously threatened by rising temperatures brought on by global warming. Moreover, the cultivable area of *P. ginseng* is gradually diminishing. Therefore, a number of physiological and morphological studies have been carried out to comprehend the high temperature response of *P. ginseng* [20,21,22]. However, to better understand how *P. ginseng* responds to high temperatures, a thorough investigation incorporating morphological, genomic, transcriptomic, and physiological analyses is still necessary.

During the domestication of root crops, plants were largely selected for their capacity to store beneficial chemicals and energy sources in their storage roots. Since *P. ginseng* requires a culture period of 4–6 years and exhibits a moderate growth rate, high temperature tolerance has a great effect on its secondary root growth and consequently is important for increasing yield [23]. The expression of genes encoding heat shock transcription factors (HSFs) and heat shock proteins (HSPs) are upregulated by high temperature and primarily regulated by plant hormones such as abscisic acid (ABA), jasmonic acid (JA), and salicylic acid (SA) [10,24,25]. Previous studies show that phytohormones and their crosstalk influence the plant response to high temperature, which damages the photosynthetic system and chloroplasts in diverse ways, ranging from a mild reduction in photosynthetic rate to permanent impairment of photosynthesis [26,27,28]. In a recent study, genome-wide transcriptional changes were evaluated and compared in two representative *P. ginseng* cultivars to better understand their response to high temperature [29]. The results showed that genes encoding WRKY transcription factors, fatty acid desaturases, and chlorophyll a/b binding proteins are important for the adaptation of *P. ginseng* to cold and shade, and their expression has a significant negative impact on heat and light tolerance. Additionally, transcript levels of genes associated with photosynthesis and sugar metabolism were significantly decreased by long-term exposure to high temperature [29]. Furthermore, proteomics investigation of *P. ginseng* leaves under heat stress revealed that the abundance of photosynthesis and phytohormone signaling related proteins was diminished, whereas that of RNA transport and ribosome biogenesis-related proteins was stimulated [30]. However, the majority of studies conducted to date on the high temperature response of *P. ginseng* focused mainly on the physiological and genetic analyses of shoots and leaves.

In general, high temperatures shorten the life cycle of plants and limit their ability to photosynthesize, which lowers agricultural production [31]. In this study, we examined the physiological effects of high temperature on the development of *P. ginseng* roots and shoots. Under extreme heat, *P. ginseng* shoots and roots exhibited drastically reduced primary and secondary growth. It has been proposed that the primary and secondary growth of high temperature-treated *P. ginseng* plants is controlled by a transcriptional network, which comprises genes involved in controlling the growth-promoting response to high temperature as well as stress-related hormones such as ABA, JA, SA, and ethylene (ET). Genes related to cell wall organization, photosynthesis, and carbohydrate biosynthesis were downregulated in response to high temperature. Additionally, the formation of starch granules in storage parenchymal cells was reduced as a result of interactions among these signaling networks. Overall, our findings offer insight into the connection between the plant response to high temperature and the mechanism regulating plant development in *P. ginseng*.

## 2. Results

### 2.1. High Temperature Retards Primary Shoot Growth in P. ginseng

**Figure 1:**
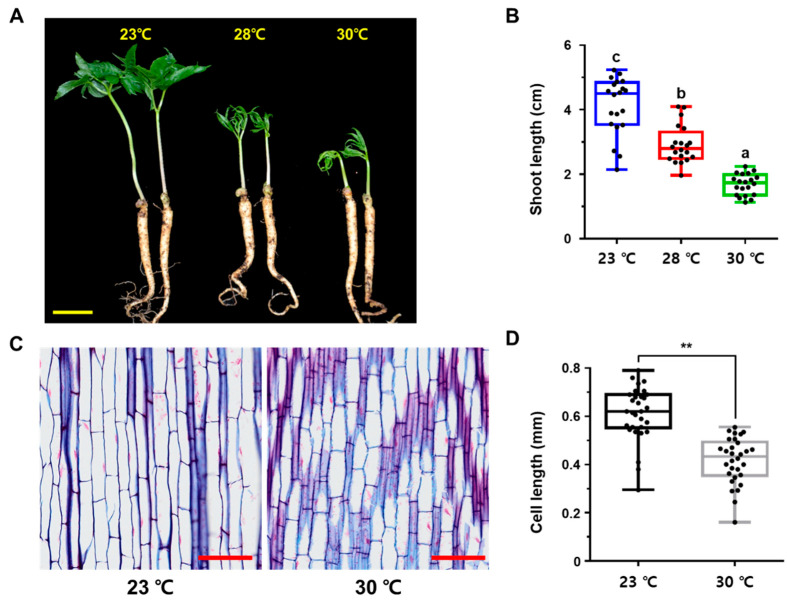
Characterization of thermomorphogenic responses in shoot and root development of *P. ginseng*. (**A**) Phenotype of 1-year old *P. ginseng* cultivar, Yunpoong, treated for one week after germination at optimal temperature (23 °C) or high temperature (28 °C or 30 °C). Scale bar = 20 mm. (**B**) Measurement of stem length of (**A**). Different lowercase letters indicate statistically significant differences *p* < 0.05; one-way analysis of variance [ANOVA], followed by Tukey’s multiple range test). (**C**) Longitudinal cross-sections of *P. ginseng* stems treated with 23 °C or 30 °C. Scale bar = 0.5 mm. (**D**) Quantification cell length in (**C**) was measured using ImageJ software (** *p* > 0.01, the significance of the difference was analyzed by *t*-test method). In (**B**,**D**), dots represent individual values. Error bars represent standard error; *n* = 20 (**B**), *n* = 30 (**D**).

Long-term exposure to high temperature typically causes dramatic changes in plant growth and development through a process known as thermomorphogenesis, which is characterized by petiole hyponasty, the inhibition of shoot and root growth, and a reduction in leaf blade size [9,10]. This phenotypic and developmental plasticity under heat stress enables plants to maximize their fitness in the changed environment. To investigate whether high temperature stress affects the growth and development of *P. ginseng* plants, 1-year-old seedlings were exposed to normal conditions (23 °C, control) and high temperatures (28 °C and 30 °C) for 1 week after rhizome germination (Figure 1). Primary shoot growth, leaf expansion, and hyponasty were retarded at both 28 °C and 30 °C (Figure 1A). The shoot lengths of plants exposed to 28 °C and 30 °C were significantly reduced by approximately 29% and 59%, respectively, compared with the control (Figure 1B). We then performed a histological analysis of paraffin-embedded sections of ginseng shoots to gain a better understanding of the physiological effects of high temperature on ginseng shoot growth. High temperature-treated shoots contained approximately 32% lower epidermal cells than control shoots (Figure 1C,D). These findings imply that primary shoot growth in *P. ginseng*, unlike that in other plant species, is sensitive to high temperature stress.

### 2.2. Transcriptome Analysis of P. ginseng Plants Exposed to High Temperatures

**Figure 2:**
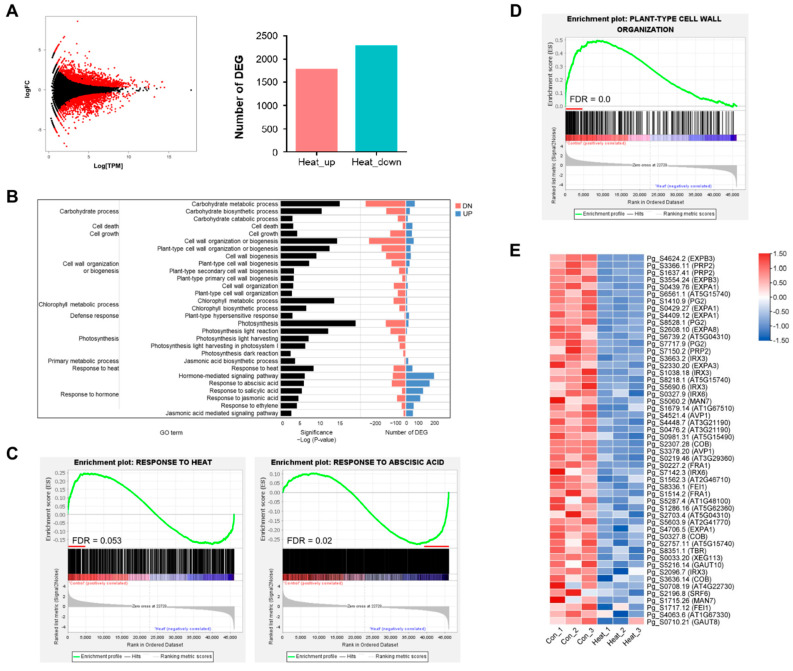
Genome-wide transcriptome profiling of *P. ginseng* treated with or without high temperature. (**A**) MA plot comparing the differential expression of control and heat-treated ginseng samples. The genes that were either upregulated (1777) or downregulated (2280) with a q value of less than 0.05 and a fold change of more than 1.5 are represented by the red dots and bar graph. (**B**) An examination of the enrichment of the gene ontology (GO) category for differentially expressed genes (DEGs), which was determined by comparing control and heat-treated samples. We chose GO terms from the biological process level 3 and level 5 categories that had an EASE score of less than 0.01 (left panel). The right panel displays the total number of genes that were either upregulated (shown in red) or downregulated (shown in green) as a result of the enriched GO terms. (**C**) Enrichment plot for a response to heat (GO:0009408) and abscisic acid (GO:0009737). (**D**) Enrichment plot for plant-type cell wall organization (GO:0009664). The red line indicates the leading-edge subset, which were selected in the enrichment plot. This line indicates the gene subset that had the greatest impact on the enrichment score (ES) and had a false discovery rate (FDR) of less than 0.05. (**E**) A heatmap depicting the expression of the leading-edge subset of genes that are associated with the plant-type cell wall organization.

To investigate the unique mechanism of thermomorphogenesis in *P. ginseng*, we reanalyzed the previously reported RNA-seq data of *P. ginseng* plants treated with prolonged heat stress [29]. A total of 4057 differentially expressed genes (DEGs; *q* < 0.05, fold change ≥ 1.5) were identified, of which 1777 were upregulated and 2280 were downregulated (Figure 2A). Gene ontology (GO) enrichment analysis of the DEGs showed that high temperature treatment was significantly associated with the following functional categories: (1) ‘carbohydrate metabolic process’ (*p*-values of sub-GO terms: *p* = 9.9 × 10^−16^ for carbohydrate metabolic process; *p* = 3.7 × 10^−11^ for carbohydrate biosynthetic process), (2) ‘cell growth’ (*p* = 4.4 × 10^−5^), ‘cell wall organization or biogenesis’ (*p* = 7.2 × 10^−10^ for cell wall biogenesis; *p* = 0.0004 for cell wall organization), (3) ‘photosynthesis’ (*p* = 1.0 × 10^−90^ for photosynthesis; *p* = 8.0 × 10^−13^ for photosynthesis, light reaction; *p* = 0.002 for photosynthesis, dark reaction), (4) ‘response to heat’ (*p* = 3.8 × 10^−9^), and (5) ‘response to hormone’ (*p* = 8.3 × 10^−7^ for response to abscisic acid; *p* = 3.4 × 10^−6^ for response to salicylic acid; *p* = 2.3 × 10^−5^ for response to jasmonic acid; *p* = 6.2 × 10^−5^ for response to ethylene) (Figure 2B). Gene set enrichment analysis (GSEA) revealed that the response to heat-related term was not significantly enriched in high temperature-treated ginseng (false discovery rate [FDR] = 0.053), whereas the response to abscisic acid was significantly enriched (FDR = 0.02) (Figure 2C). In terms of expression variations, we discovered 81 and 165 key leading-edge subset genes that were enriched gene set groups and produced enrichment scores (ES) (Figure S1A,B and Table S1). The expression levels of genes that respond to heat were determined using RNA-seq data and validated by quantitative real-time PCR (qRT-PCR) (Figure S1C). Additionally, GSEA demonstrated that the leading-edge subset identified in high temperature-treated *P. ginseng* plants was composed of genes activated by ET, SA, and JA (Figure S2). 

Next, we focused on the functional enrichment analysis of genes related to ‘plant type-cell wall organization’ in high temperature-treated ginseng. GESA was used to validate the significantly enriched GO terms, including ‘plant type-cell wall organization’, ‘plant type-cell wall biogenesis’, and ‘cell growth’ (Figure 2D and Figure S3A,B). A total of 52 genes were found to comprise a critical leading-edge subset of the enriched gene set group in the GSEA (Figure 2E and Figure S3C,D). These leading-edge subset genes, including *EXPANSIN* (*EXP*) genes (*EXPA*s and *EXPB*s), were significantly downregulated in the high temperature-treated ginseng samples. Subsequently, the expression pattern of DEGs related to plant-type cell wall organization was confirmed by real-time qRT-PCR (Figure S4). These findings reveal that high temperature controls shoot elongation by regulating the structure and biogenesis of the cell wall.

### 2.3. High Temperature Retards Root Secondary Growth in P. ginseng

**Figure 3:**
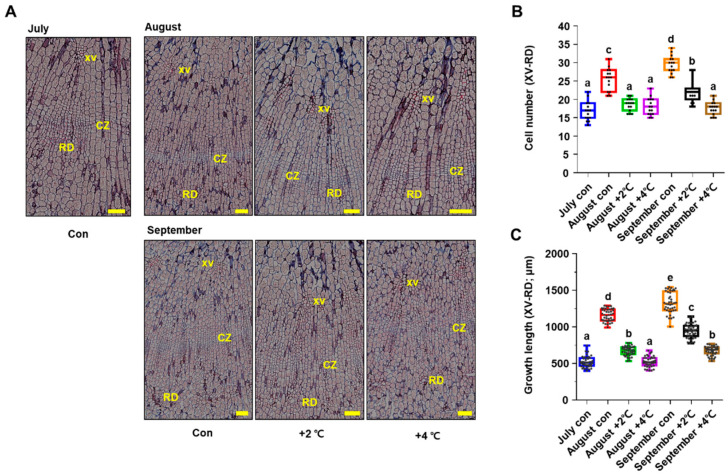
High temperature treatment represses root secondary growth in *P. ginseng*. (**A**) Representative root images of stained stem cross-sections of *P. ginseng* plants treated with a mock control (Con) or high temperature (+2 °C or +4 °C) from July to September. XV: xylem vessel, CZ: cambial cell layer zone, RD: resin duct cells. Scale bar = 200 μm. (**B**) Quantification of cambium-derived cells in XV and RD of each ray. (**C**) Quantification cell length in (**A**) was measured using ImageJ software. Dots represent individual values. Different lowercase letters indicate statistically significant differences *p* < 0.05. Error bars represent standard error; *n* = 15 (**B**), 41 (**C**). *p* < 0.05; one-way ANOVA.

RNA-seq results further revealed that long-term heat stress greatly reduced photosynthesis and carbohydrate metabolism (Figure 2B). This led us to hypothesize that heat-stress-induced reduction in photosynthesis has deleterious effects on the development of storage organs in *P. ginseng*. To gain insight into the physiological effects of high temperatures on root secondary growth in *P. ginseng*, we performed a histological analysis of paraffin-embedded sections of roots (Figure 3). Initially, we monitored the growth phenotypes of dividing meristematic stem cells in the cambial zone (CZ) and the size of divided cells surrounding the cambium layer of the storage tap roots from July to September under optimal conditions (control, Figure 3). The number and length of divided starch-deposited storage parenchymal cells located between the xylem vessels (XVs) and resin ducts (RDs) increased significantly in the control treatment from July to September (Figure 3B,C). However, ginseng samples treated with high temperature for 1 or 2 months in the soil-plant-atmosphere-research (SPAR) chamber showed a significant reduction in the number and length of cambium-derived cells in the XVs and RDs. Additionally, high temperature-treated ginseng roots exhibited approximately 42% and 55% shorter XV–RD length than mock-treated roots at +2 °C and +4 °C, respectively, in August (Figure 3C). In September, the XV–RD length was shortened by 29% and 49% in high temperature-treated roots at +2 °C and +4 °C, respectively, compared with mock-treated roots. Moreover, high temperature-treated ginseng roots showed a greater reduction in the number and length of cells at +4 °C than at +2 °C compared with the control, suggesting that secondary root growth in *P. ginseng* decreases with increasing temperature (Figure 3). The results of phenotypic analysis were consistent with those of transcriptome analysis (Figure 2 and Figure 3).

### 2.4. High Temperature Negatively Regulates Both the Light and Dark Reactions of Photosynthesis

**Figure 4:**
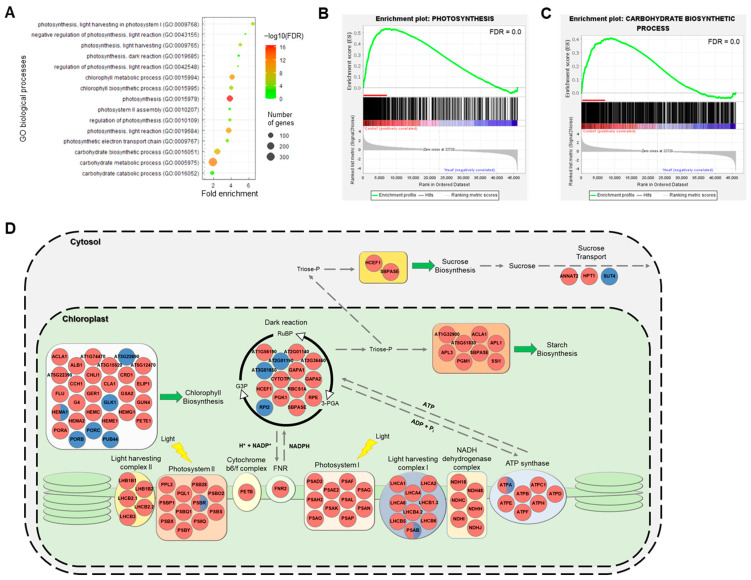
Functional enrichment of photosynthesis and carbohydrate related terms in the high temperature-mediated growth retardation of *P. ginseng*. (**A**) Photosynthesis and carbohydrate related biological processes of DEGs are represented and are sorted by fold enrichment. The dot size indicates the number of DEGs associated with the process and the dot color indicates the significance of the enrichment (−log10 (FDR-corrected *p*-values)). The vertical grey dashed line represents a fold enrichment of 1. (**B**,**C**) Enrichment plot for photosynthesis (GO:0015979) and carbohydrate biosynthesis process (GO:0016051). Red lines indicate the leading-edge subset genes in the GSEA. (**D**) Diagram of the photosynthesis pathway among the upregulated (blue circle) and downregulated (red) genes involved in photosynthesis. Red/blue semicircle indicated that all of the upregulated and downregulated genes in *P. ginseng* were included. The list of genes is in Table S2. Thick gray dashed lines indicate membrane.

**Figure 5:**
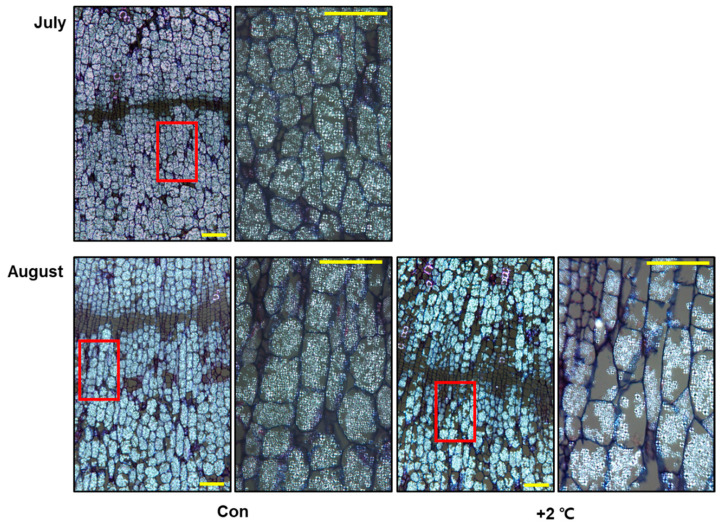
High temperature negatively regulates storage parenchymal cell development in ginseng roots. Starch granule development in storage parenchymal cells of *P. ginseng* tap roots grown with a control (Con) or high temperature (+2 °C) from July to August. Scale bar = 100 µm.

Among the GO processes affected by high temperature treatment, we focused on the highly significantly downregulated processes: ‘carbohydrate process’, ‘chlorophyll process’, and ‘photosynthesis process’. Following GO enrichment analysis, we identified the significant pathway (FDR < 0.05) of GO terms related to photosynthesis (Figure 4A). The enriched terms included ‘photosynthesis light harvesting’, ‘photosynthesis light reaction’, photosynthesis dark reaction’, ‘chlorophyll biosynthetic process’, ‘carbohydrate biosynthetic process’. We performed the GSEA to identify the major terms related to the leading-edge subset genes (Figure 4B,C and Figure S5). We found that chlorophyll biosynthesis, photosynthesis, and carbohydrate biosynthetic process–related terms were significantly enriched in the GSEA (Figure 4B,C and Figure S5). In addition, our data showed that GO terms related to the light and dark reactions of photosynthesis and those related to starch biosynthesis were negatively correlated with the long-term high temperature stress treatment (Figure S6). These findings suggest that defects in photosynthesis and its ensuing metabolic processes are strongly linked with high temperature-mediated growth and developmental retardation. To further investigate this idea more specifically, we confirmed the expression patterns of DEGs encoding chloroplast-localized photosynthetic proteins and cytosol-localized sucrose biosynthesis and transport related proteins (Figure 4D). The selected DEGs, which were picked based on their similarity to Arabidopsis genes, were primarily downregulated by prolonged exposure to high temperature in the major pathways of the light and dark reactions of photosynthesis, starch biosynthesis, and sucrose transport (Figure 4D). These finding led us to hypothesize that high temperature treatment retards the development of starch granules in storage parenchymal cells in the main tap root. We monitored the development of starch granules in high temperature-treated ginseng roots for 1 month (Figure 5). The results demonstrated that high temperature treatment repressed the differentiation of storage parenchymal cells and the production of starch granules in these cells (Figure 5).

**Figure 6:**
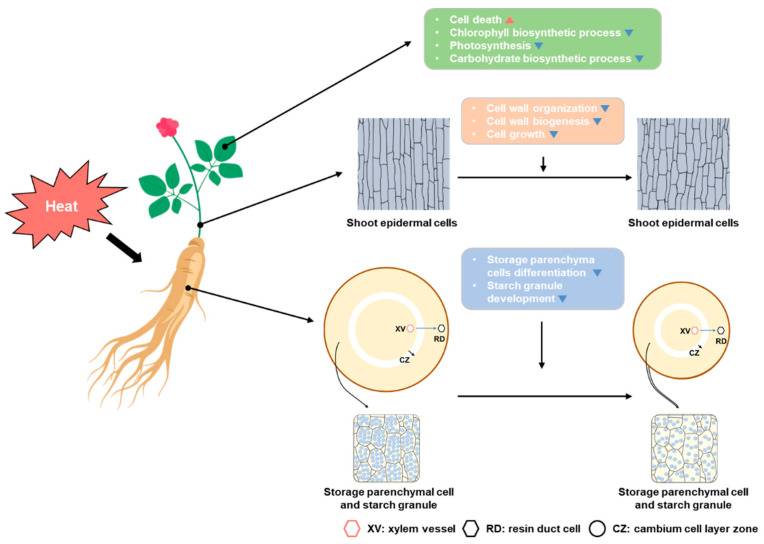
Schematic model of the effects of high temperatures on *P. ginseng*. High temperatures can alter or damage the function or activity of leaf cells and tissue, such as cell wall organization, photosynthesis, and chlorophyll biosynthesis. In cells of the stem, it has effects related to cell wall organization, biogenesis, and cell growth. Then, high temperature affects the root secondary growth and the development of starch granules in the storage parenchymal cells.

Taken together, our findings reveal that prolonged exposure to high temperature is detrimental to the growth and development of *P. ginseng*. High temperature appears to be recognized primarily in the shoot and induces the retardation of stem growth and leaf expansion by repressing the expression of cell wall organization and biogenesis as well as cell growth related genes such as *EXPs*. Furthermore, high temperature activates stress-related hormones and reactive oxygen species (ROS) signaling pathways and gradually reduces photosynthetic activity. These effects inhibit carbohydrate biosynthesis and subsequently diminish phloem transport, decreasing the sink activity of ginseng storage roots. In addition, we discovered that cambium activity was reduced in storage roots, and the differentiation of storage parenchymal cells and the development of starch granules were inhibited by the extended exposure to high temperature (Figure 6).

## 3. Discussion

Global warming has increased the frequency of heat waves and tropical nights in recent years [5,6,8]. Growth of ginseng is frequently inhibited by biotic and abiotic stresses, with high temperature being the most detrimental environmental stress. In the last decade, the molecular genetic mechanisms of plant physiological responses to high temperature have been studied in model plants including Arabidopsis, rice, and tomato [9,32]. However, only a few studies have been conducted in *P. ginseng* to investigate the regulatory networks involved in the plant response to high temperature [29,30]. Some genes may have functionally diverged over the course of evolution, and homologous genes in other plant species may have evolved to perform different functions. Therefore, additional research on *P. ginseng* is necessary to enhance our understanding of the regulatory mechanisms involved in the response to high temperatures. The response of *P. ginseng* to high temperature and the integration of signaling pathways with the downstream signaling network that controls plant growth and development need to be elucidated. In this study, the physiological roles of the high temperature response of *P. ginseng* in shoot and root growth retardation were investigated, and the core transcriptional network involved in this process was elucidated (Figure 6). Our results demonstrated that high temperature inhibits the primary and secondary growth of *P. ginseng* shoots and roots, respectively (Figure 1 and Figure 3). In addition, high temperatures inhibited the development of starch granules in storage parenchymal cells by downregulating photosynthesis and carbohydrate biosynthesis-related genes (Figure 4 and Figure 5).

To date, extensive research has been conducted on the complex hormonal regulation of plant growth at high temperature [10,32]. Studies show that plant hormones such as ABA, JA, SA, and ET contribute to thermotolerance by reducing oxidative damage and providing physiological protection from high temperature-induced damage [33,34,35,36]. The results of RNA-seq data analysis and GESA showed that high temperature enhanced the expression of *P. ginseng* genes involved in the response to ABA, JA, SA, and ET (Figure 2 and Figure S2). These results are consistent with those of previous studies in other plant species [19]. Further research is needed to determine the function of these genes and other downstream genes involved in the response to well-known stress hormones (ABA, JA, SA, and ET) in *P. ginseng*.

Histological analysis of shoot and root sections was performed to understand the high temperature-induced changes in plant growth and development in *P. ginseng* (Figure 1 and Figure 3). Transcriptome profiling of *P. ginseng* suggests that high-temperature-repressed shoot and root growth is associated with cell wall organization and biogenesis (Figure 2 and Figure S3). The remodeling of plant cell wall composition, which is highly flexible and diverse in nature, is critical during plant growth [37,38,39]. The cell wall is the first physical barrier that protects plants against heat stress. The modification of cell wall structure is important as it improves the ability of plants to perceive and respond to multiple abiotic stresses, thus conferring stress tolerance [40]. In addition, studies show that plants respond to high temperature stress through cell wall loosening [41,42,43]. However, in the current study, high temperature treatment retarded the growth of *P. ginseng* shoots and roots by repressing cell wall loosening (Figure 1 and Figure 3). Furthermore, our RNA-seq data analysis and GSEA results revealed that high temperature decreased the expression of genes related to cell wall loosening and biogenesis (Figure 2, Figures S3 and S4). These results suggest that the genetic and physiological effects of high temperature on plant growth differ between sun and shade plants. Additionally, these findings indicate that the formation of a signaling network through plant hormone crosstalk is important for the high temperature response and growth in *P. ginseng*. This further emphasizes the importance of understanding the response to and mechanisms of development under high temperature stress in *P. ginseng*.

GO enrichment analysis revealed that the GO term ‘photosynthesis’ was significantly enriched in the biological process category (Figure 2B and Figure 4A). Genes related to light harvesting and the light and dark reactions of photosynthesis were significantly selected in the GSEA (Figure S6), and many photosynthesis-related genes were differentially regulated in high temperature-treated ginseng (Figure S5). Many previous studies have shown that photosynthesis is extremely sensitive to high temperature and is inhibited long before the impairment of photosystems [3,44]. Accordingly, the expression patterns of photosynthesis-related genes (light harvesting, light reaction, dark reaction, carbohydrate, and chlorophyll) were reduced in high temperature-treated ginseng plants (Figure 4D and Figure S5). Consistently, high temperature treatment appeared to repress starch granule development and reduce shoot and root growth in ginseng (Figure 5). In addition, starch is the key determinant of plant fitness under abiotic stress conditions such as high temperature [45,46]. These results suggest that the inhibition of photosynthesis and plant fitness at high temperature represses the production of starch granules in storage parenchymal cells of *P. ginseng* roots (Figure 5).

## 4. Materials and Methods

### 4.1. Plant Materials and Growth Condition

One-year-old *P. ginseng* Yunpoong cultivar seedlings were provided by the National Institute of Horticultural and Herbal Science of Korea. To test thermomorphogenic responses of *P. ginseng*, the seedlings were germinated for 3 days at 23 °C with a 16 h light/8 h dark cycle into the ginseng cultivation soil medium in a greenhouse. The fully germinated seedlings were transferred into growth chambers at 23, 28, and 30 °C for 7 days. To investigate the response to prolonged high temperature exposure, on 22April 2021, the seedlings were transplanted in plastic pots with a mixture of commercial horticultural medium soil (Golden Root; Nongkyung Co., Jincheon, Korea). They were transferred to the SPAR (soil-plant-atmosphere research) chambers at the Department of Herbal Crop Research in Eumseong, South Korea (127°45′13.14 E, 36°56′36.63 N), where a four-layered shade net was placed over them. To prepare appropriate heat-treated ginseng root samples, 1-year-old seedlings were placed in SPAR chamber from June to September of 2022. The temperature in the SPAR chamber was 2 °C, 4 °C, and 6 °C higher than the 10-year (2012 to 2021) hourly average temperature of Eumsung, South Korea. The chambers’ relative humidity and CO_2_ concentrations were set to 60% and 400 ppm, respectively, and an auto-dripper irrigation system was used to maintain the soil water content at about 15–20%. With information from the Korea Meteorological Administration, the average hourly temperatures in Eumseong during the previous 10 years (2011–2020) were determined. From June through August, four ETC treatments were applied to the plants in the SPAR chambers: 0 °C (control treatment) and 2 °C above the mean hourly average air temperature. 

### 4.2. Histological Sections and Microscopy

We used paraffin-mediated sectioning to obtain histological section images of *P. ginseng* root and shoot samples. The paraffin sectioning method was carried out in the same manner as described in our previous work [23]. Fresh *P. ginseng* roots and shoots were fixed in 1% glutaraldehyde and 4% formaldehyde in PBS pH 7.0 overnight at 4 °C. The tissues were dehydrated in 30%, 50%, 70%, 90%, and 100% EtOH three times for one hour each before being embedded in paraffin. Microtome sections of 5–10 µm thickness were mounted on slides and stained with 1% Safranin-O (Cat. S2255, SIGMA, St. Louis, MO, USA) and 0.5% Astra blue (Cat. sc-214558A, Santa-Cruz Biochem, Dallas, TX, USA). Using a Slideview scanner and a BX53 microscope, bright and polarized light images of *P. ginseng* samples were obtained (Olympus, Tokyo, Japan). The number of root cells was counted along a straight line drawn by cambium layers from resin ducts to inner xylem vessel cells to compare the secondary growth of storage roots. 

### 4.3. RNA Extraction and qRT-PCR

Total RNA was extracted from *P. ginseng* shoots treated with mock and heat stress for 4 and 7 days, respectively, using an Easy Spin RNA Extraction Kit (iNtRON Biotechnology, Seongnam, Korea) according to the manufacturer’s instructions. cDNA was produced with the TOP scriptTM RT Dry MIX to confirm the RNA-seq data (Cat. no. RT200, Enzynomics, Daejeon, Korea). Quantitative real-time reverse transcription-polymerase chain reaction (qRT-PCR) was carried out with a KOD SYBR qRT MIX (TOYOBO, Osaka, Japan) to validate the transcripts level of Gene Set Enrichment Analysis (GSEA) transcriptome data that is down or upregulated by heat stress. The internal control for qRT-PCR was PgACT. All primer sequences are presented in Table S3.

### 4.4. Differentially Expressed Genes Analysis

Using prinseq-lite version (0.20.4) and the following parameters: min len 50; min qual score 5; min qual mean 15; derep 14; trim qual left 15; trim qual right 15 [47]. Using Bowtie 2 [48], each sample’s paired-end reads were aligned to the ginseng reference sequences. For each transcript, read counts and TMM-normalized TPM (trimmed mean of M value-normalized transcripts per million) values were obtained using the RSEM 1.3.0 program [49]. EdgeR version 3.16.5 was used to identify differentially expressed genes by calculating the negative binomial dispersion across conditions for differential gene expression analysis [50]. Significantly differentially expressed genes were considered to have a false discovery rate (FDR)-adjusted *p* value of 0.05 [51]. The Ginseng Genome Database (http://ginsengdb.snu.ac.kr/index.php, accessed on 26 February 2022) was used for reference sequences of ginseng. 

### 4.5. Functional Annotation

For functional annotation of DEGs, the BLAST program was used with e-value threshold of 1E–5 against *Arabidopsis thaliana* protein database. The DAVID platform was used for gene ontology (GO) term enrichment analysis, and the enriched GO term was determined by modified Fisher Exact test (*p* < 0.05) [52,53]. GSEA was used to further analyze enriched GO genes, as described in our previous work [51]. The bar graph was visualized with the ‘ggplot2’ R package [54]. In the enrichment plot, the red line represents the subset of genes that contributed the most to the enrichment score (ES) as the leading-edge group. In the plot, the ranking list metric measures the association between a gene and the plant phenotype. In the ranking list, positive and negative values indicate genes upregulated (red color gradient) and downregulated (blue) in control samples, respectively. To identify the most representative GO term from the list of enriched photosynthesis-related terms, we used REVIGO (http://revigo.irb.hr/, accessed on 3 July 2022) with a strict dispensability cutoff (0.05). Visualization of enriched GO terms was performed using the R package ‘ggplot2’ [55].

## 5. Conclusions

Understanding the growth and fitness of *P. ginseng* under high temperature stress can extend our knowledge of the mechanisms of high temperature responses in *P. ginseng*. Although this study demonstrates that high temperature reduces shoot and root growth and inhibits starch granule production in *P. ginseng*, the downstream signaling pathways that interact with the upstream plant hormone signaling pathways to regulate plant growth and fitness at high temperature remain unclear. Identification of the molecular mechanisms underlying the crosstalk between high temperature response-related genes and other signaling pathways during the growth and development of *P. ginseng*, a shade plant, is expected to facilitate the breeding and development of high-temperature-tolerant cultivars.

## Data Availability

The raw data were deposited in the NCBI Short Read Archive (SRA) database under the accession number PRJNA266907.

## Supplementary Materials

The following supporting information can be downloaded at: https://www.mdpi.com/article/10.3390/ijms231911647/s1.
