# Supplementary material for: Prolonged Exposure to High Temperature Inhibits Shoot Primary and Root Secondary Growth in Panax ginseng
Source: Int J Mol Sci. 2022 Oct 1;23(19):11647. doi: 10.3390/ijms231911647 (PMC9569605; doi:10.3390/ijms231911647)
Supplement: Supplementary file 1 [file ijms-23-11647-s001.zip › SuplementaryFigures.pptx]

## Slide 1
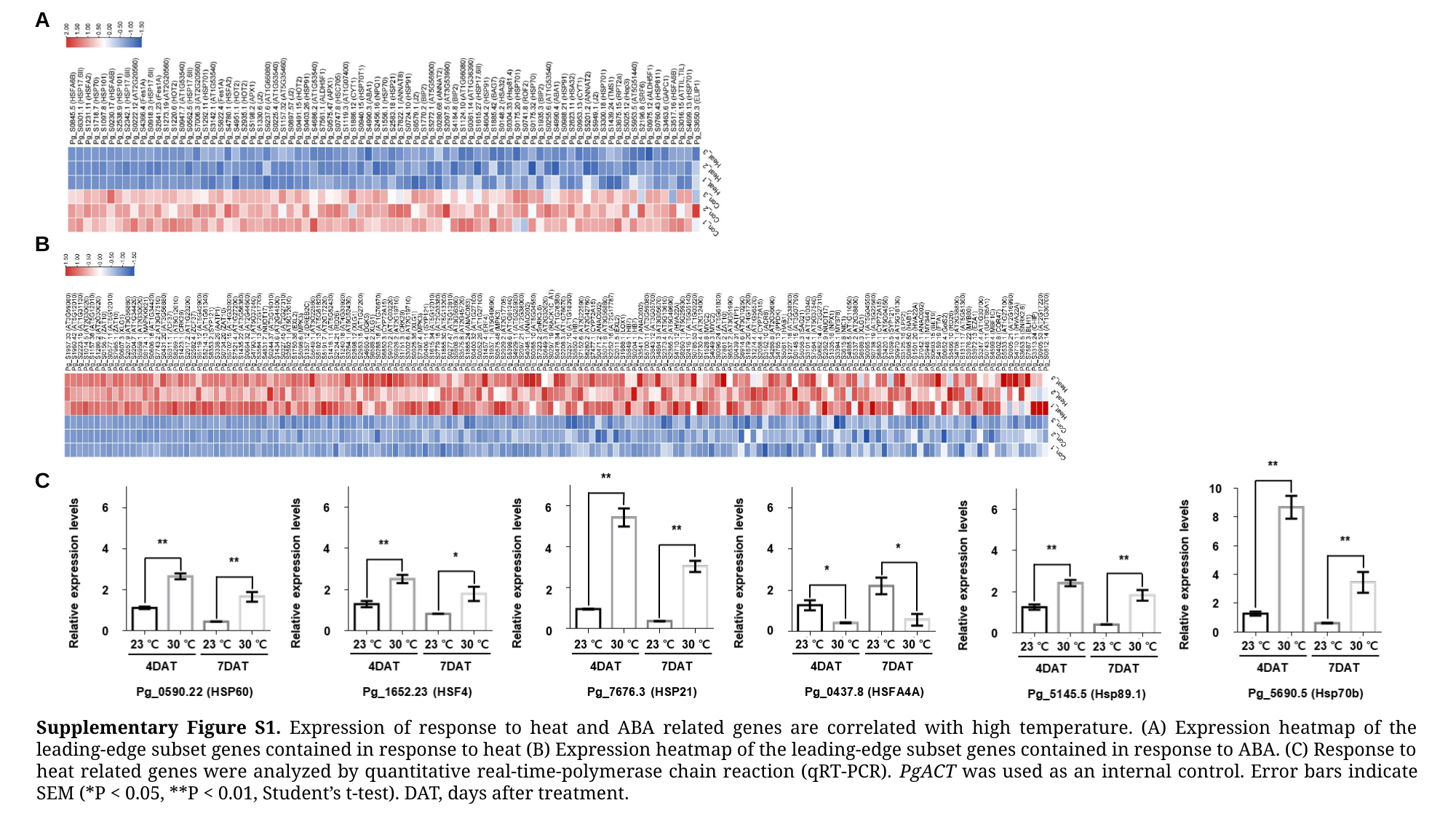

A
B
C
Supplementary Figure S1. Expression of response to heat and ABA related genes are correlated with high temperature. (A) Expression heatmap of the leading-edge subset genes contained in response to heat (B) Expression heatmap of the leading-edge subset genes contained in response to ABA. (C) Response to heat related genes were analyzed by quantitative real-time-polymerase chain reaction (qRT-PCR). PgACT was used as an internal control. Error bars indicate SEM (*P < 0.05, **P < 0.01, Student’s t-test). DAT, days after treatment.

## Slide 2
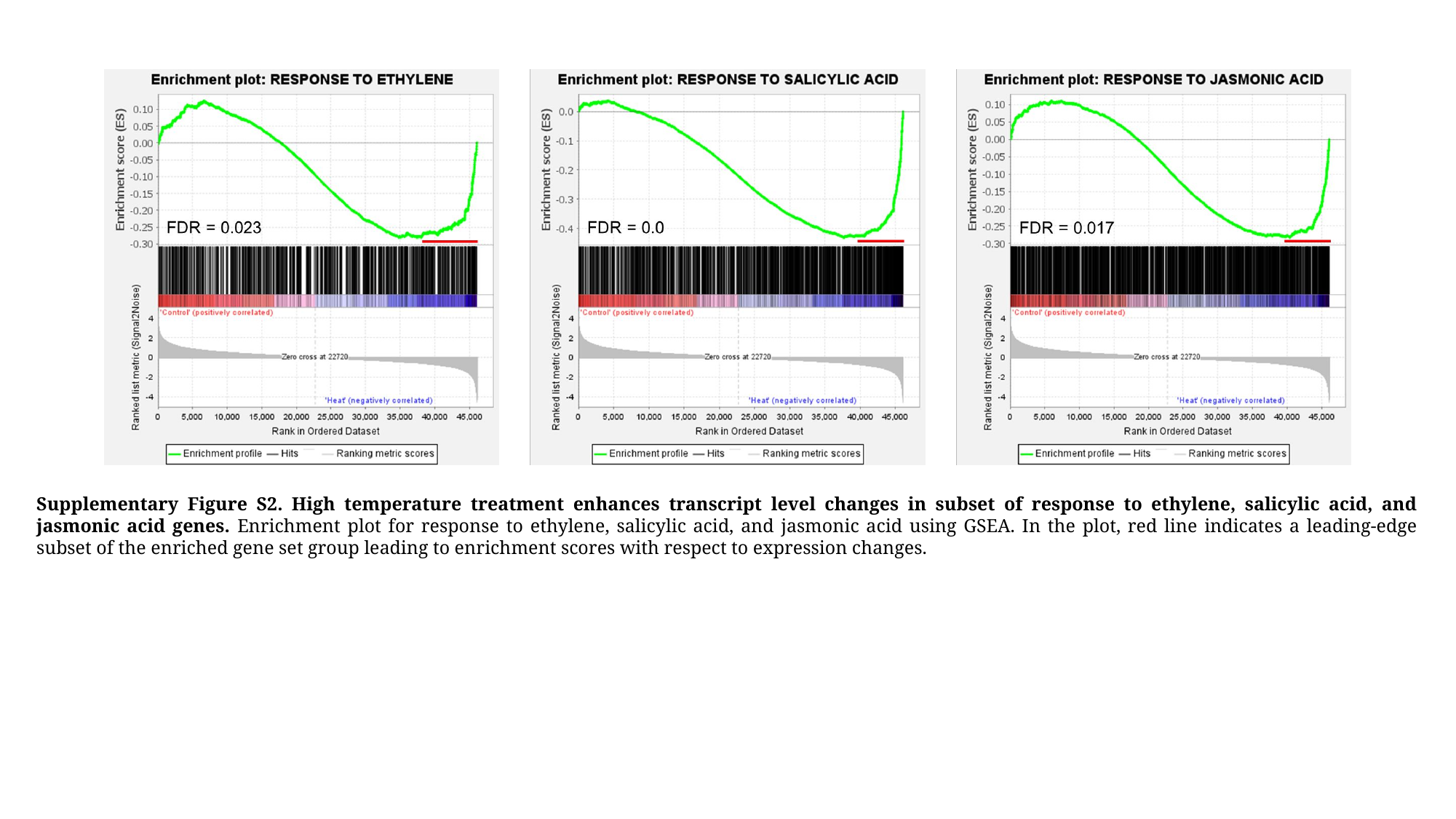

Supplementary Figure S2. High temperature treatment enhances transcript level changes in subset of response to ethylene, salicylic acid, and jasmonic acid genes. Enrichment plot for response to ethylene, salicylic acid, and jasmonic acid using GSEA. In the plot, red line indicates a leading-edge subset of the enriched gene set group leading to enrichment scores with respect to expression changes.

## Slide 3
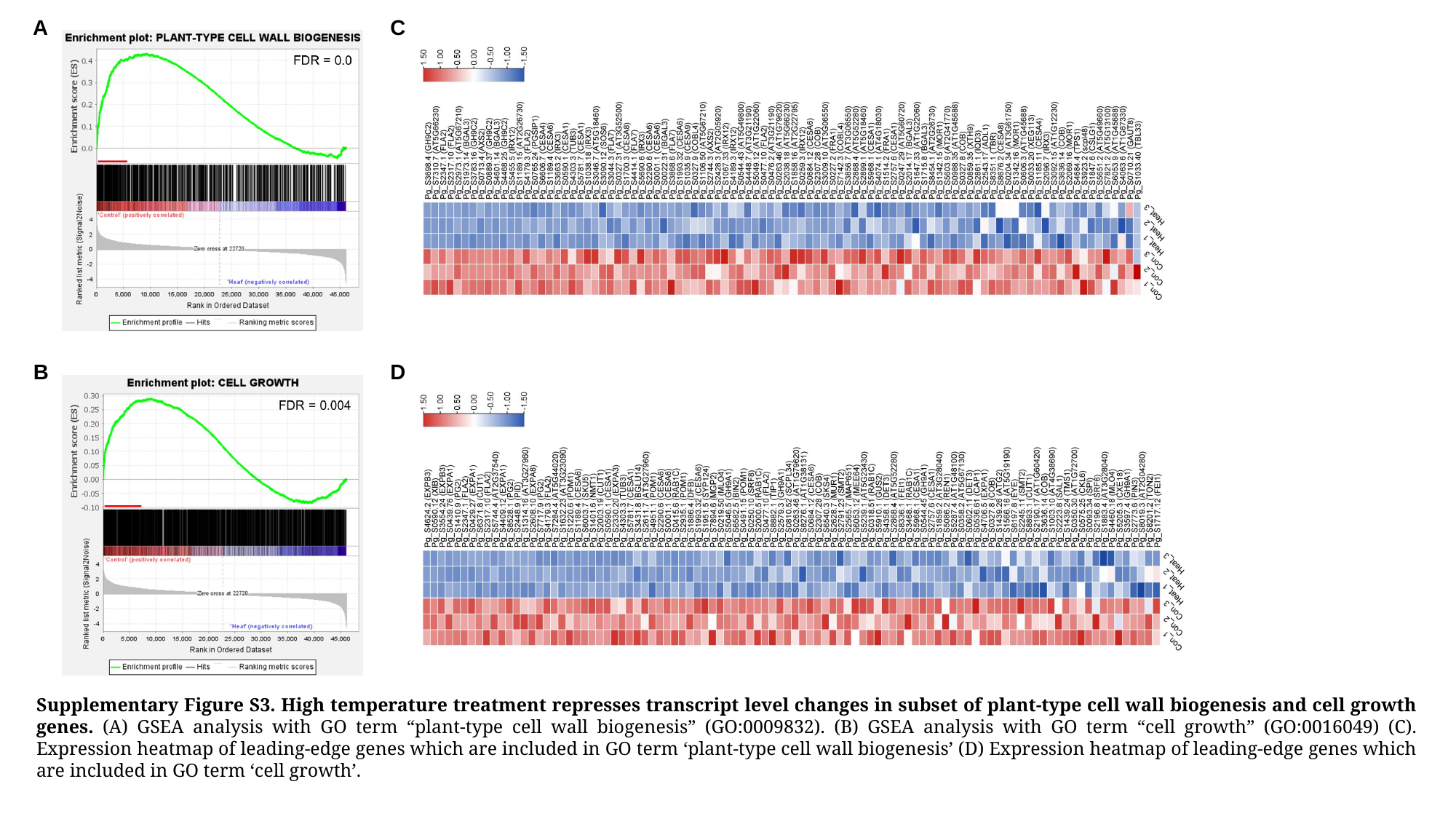

C
A
D
B
Supplementary Figure S3. High temperature treatment represses transcript level changes in subset of plant-type cell wall biogenesis and cell growth genes. (A) GSEA analysis with GO term “plant-type cell wall biogenesis” (GO:0009832). (B) GSEA analysis with GO term “cell growth” (GO:0016049) (C). Expression heatmap of leading-edge genes which are included in GO term ‘plant-type cell wall biogenesis’ (D) Expression heatmap of leading-edge genes which are included in GO term ‘cell growth’.

## Slide 4
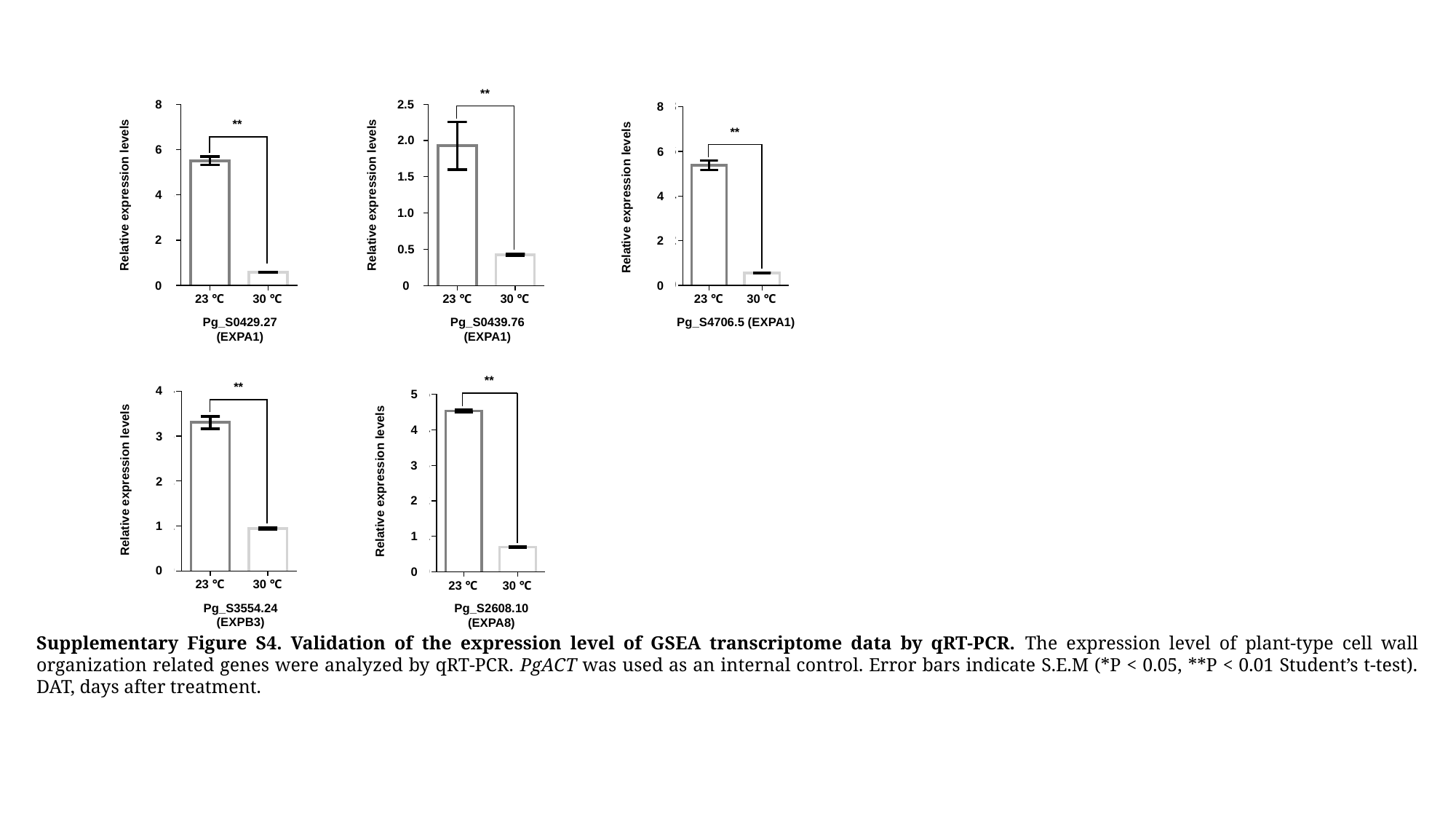

**
8
2.5
8
**
**
2.0
6
6
1.5
4
Relative expression levels
Relative expression levels
4
Relative expression levels
1.0
2
2
0.5
0
0
0
23 ℃
30 ℃
23 ℃
30 ℃
23 ℃
30 ℃
Pg_S0429.27 (EXPA1)
Pg_S0439.76 (EXPA1)
Pg_S4706.5 (EXPA1)
**
**
4
5
4
3
3
Relative expression levels
2
Relative expression levels
2
1
1
0
0
23 ℃
30 ℃
23 ℃
30 ℃
Pg_S3554.24 (EXPB3)
Pg_S2608.10 (EXPA8)
Supplementary Figure S4. Validation of the expression level of GSEA transcriptome data by qRT-PCR. The expression level of plant-type cell wall organization related genes were analyzed by qRT-PCR. PgACT was used as an internal control. Error bars indicate S.E.M (*P < 0.05, **P < 0.01 Student’s t-test). DAT, days after treatment.

## Slide 5
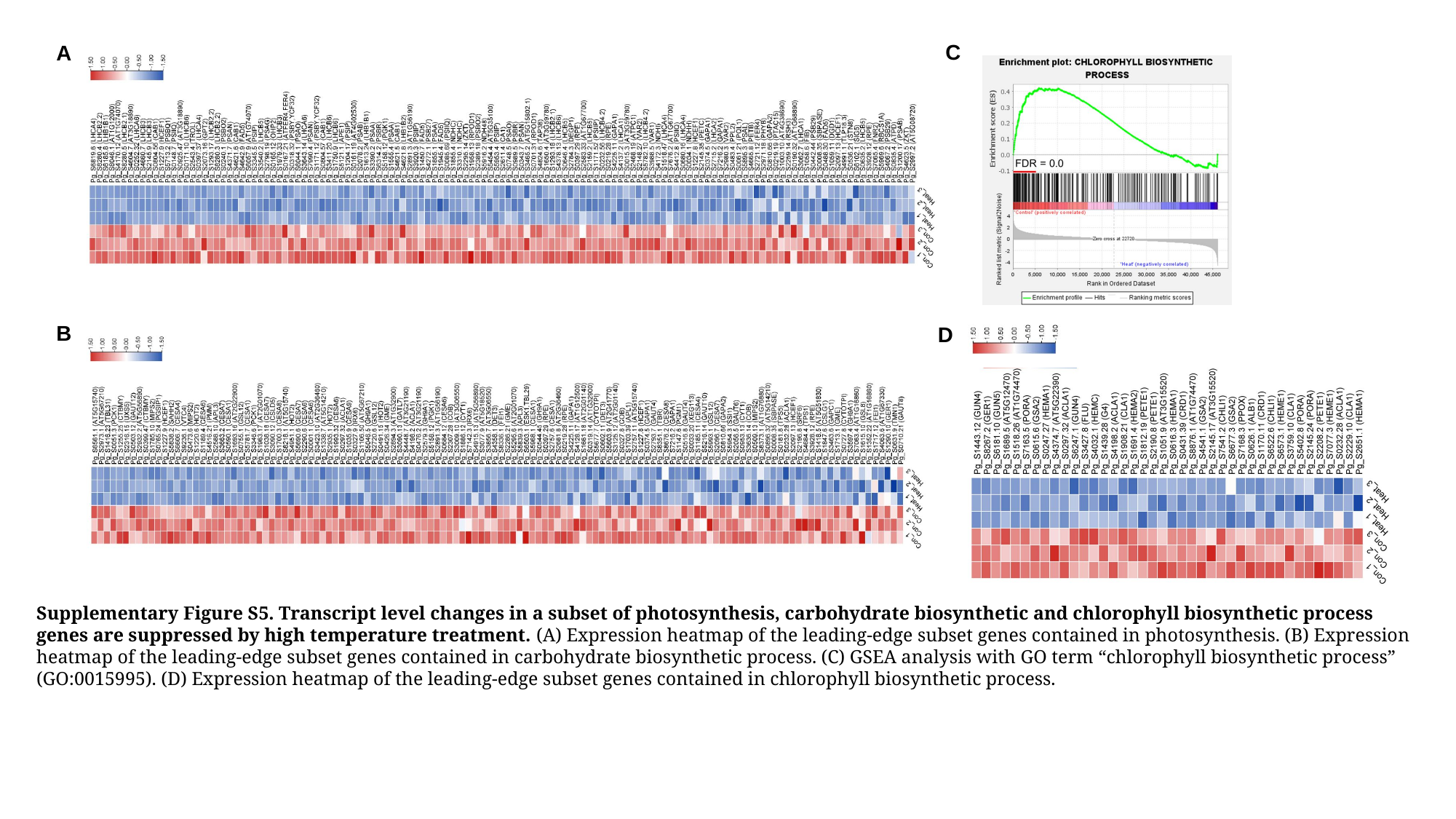

C
A
B
D
Supplementary Figure S5. Transcript level changes in a subset of photosynthesis, carbohydrate biosynthetic and chlorophyll biosynthetic process genes are suppressed by high temperature treatment. (A) Expression heatmap of the leading-edge subset genes contained in photosynthesis. (B) Expression heatmap of the leading-edge subset genes contained in carbohydrate biosynthetic process. (C) GSEA analysis with GO term “chlorophyll biosynthetic process” (GO:0015995). (D) Expression heatmap of the leading-edge subset genes contained in chlorophyll biosynthetic process.

## Slide 6
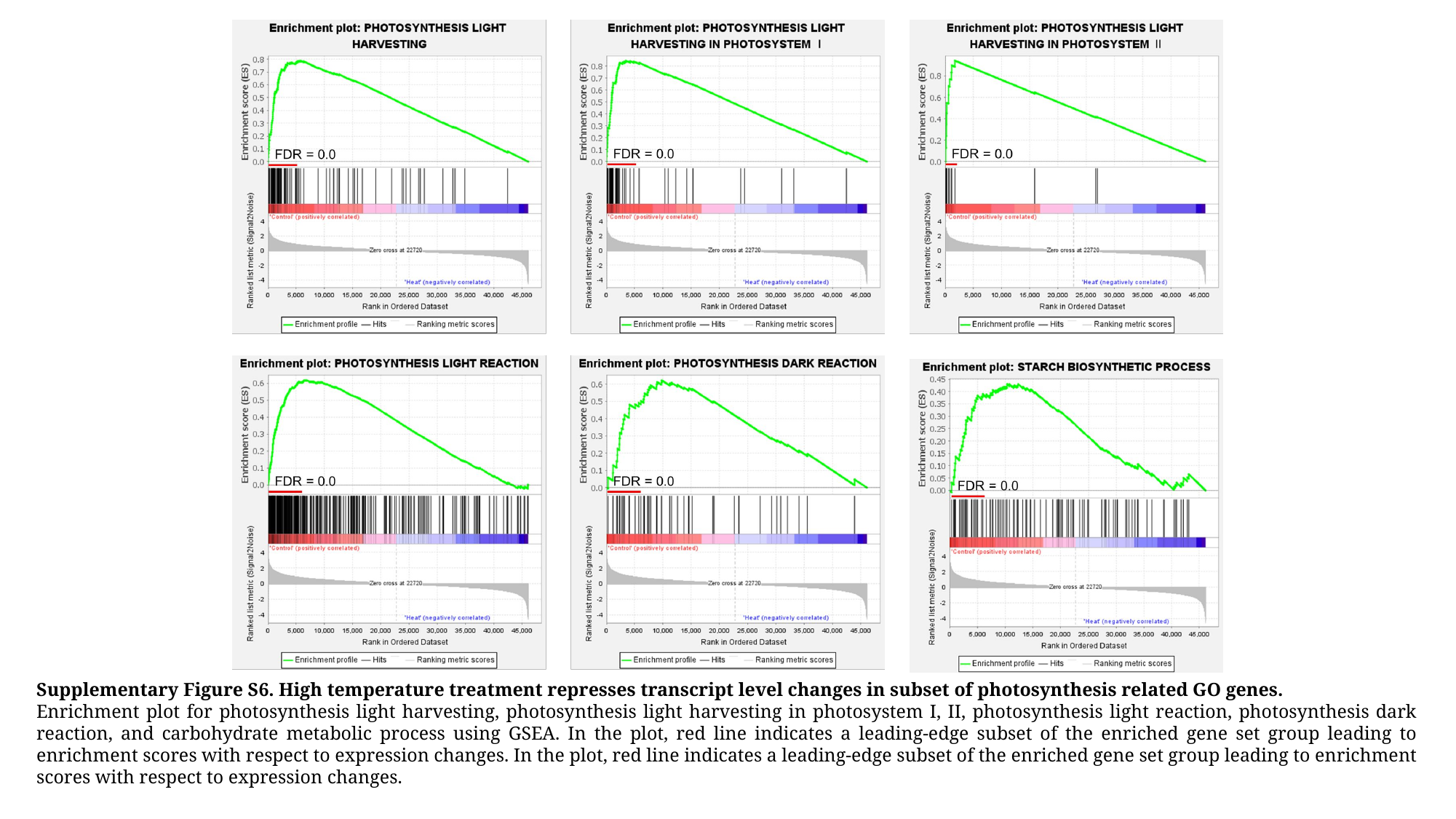

Supplementary Figure S6. High temperature treatment represses transcript level changes in subset of photosynthesis related GO genes.
Enrichment plot for photosynthesis light harvesting, photosynthesis light harvesting in photosystem I, II, photosynthesis light reaction, photosynthesis dark reaction, and carbohydrate metabolic process using GSEA. In the plot, red line indicates a leading-edge subset of the enriched gene set group leading to enrichment scores with respect to expression changes. In the plot, red line indicates a leading-edge subset of the enriched gene set group leading to enrichment scores with respect to expression changes.
